# Genomic characterization of two ranavirus isolates identified from a gopher frog (*Lithobates capito*) and a striped newt (*Notophthalmus perstriatus*) during a mass mortality event in Florida

**DOI:** 10.1128/mra.00017-24

**Published:** 2024-04-23

**Authors:** Arik Hartmann, Kuttichantran Subramaniam, Cody Conrad, Pedro H.O. Viadanna, Thomas B. Waltzek, Ana V. Longo

**Affiliations:** 1 Department of Biology, University of Florida, Gainesville, Florida, USA; 2 Department of Infectious Diseases and Immunology, College of Veterinary Medicine, University of Florida, Gainesville, Florida, USA; 3 Emerging Pathogens Institute, University of Florida, Gainesville, Florida, USA; 4 Laboratory of Viral Diseases, National Institute of Allergy and Infectious Diseases, National Institutes of Health, Bethesda, Maryland, USA; 5 School of Biological Sciences, Washington State University, Pullman, Washington, USA; 6 Department of Veterinary Microbiology and Pathology, Washington Animal Disease Diagnostic Laboratory, College of Veterinary Medicine, Washington State University, Pullman, Washington, USA; Katholieke Universiteit Leuven, Leuven, Belgium

**Keywords:** *Iridoviridae*, *frog virus 3*, salamanders, frogs, genome

## Abstract

Two ranavirus isolates were recovered from anuran and salamander samples collected during an amphibian mass mortality event in North-Central Florida in 2021. Phylogenetic analyses of the full genomes confirmed that the two isolates were nearly identical and strains of the species *Frog virus 3*.

## ANNOUNCEMENT

A larval gopher frog (*Lithobates capito*, animal ID: LC3C) and post-metamorphic striped newt (*Notophthalmus perstriatus*, animal ID: NP2C) exhibiting ulceration of mouthparts consistent with ranaviral disease were collected at the same site during a mass mortality event of wild amphibians in Florida in 2021 (1). Infection by frog virus 3 (FV3; *Iridoviridae*) was confirmed using quantitative PCR assays (2), and histopathological staining of individuals from the outbreak revealed necrosis in liver, spleen, and kidney tissues (1). *Frog virus 3* is the type species of the iridovirid genus *Ranavirus* and can infect all classes of ectothermic vertebrates (3). This work was approved by the Florida Fish and Wildlife Conservation Commission (FWC-LSSC-17-00031B) and the UF Institutional Animal Care and Use Committee (IACUC Protocol Number 201810502).

**Fig 1:**
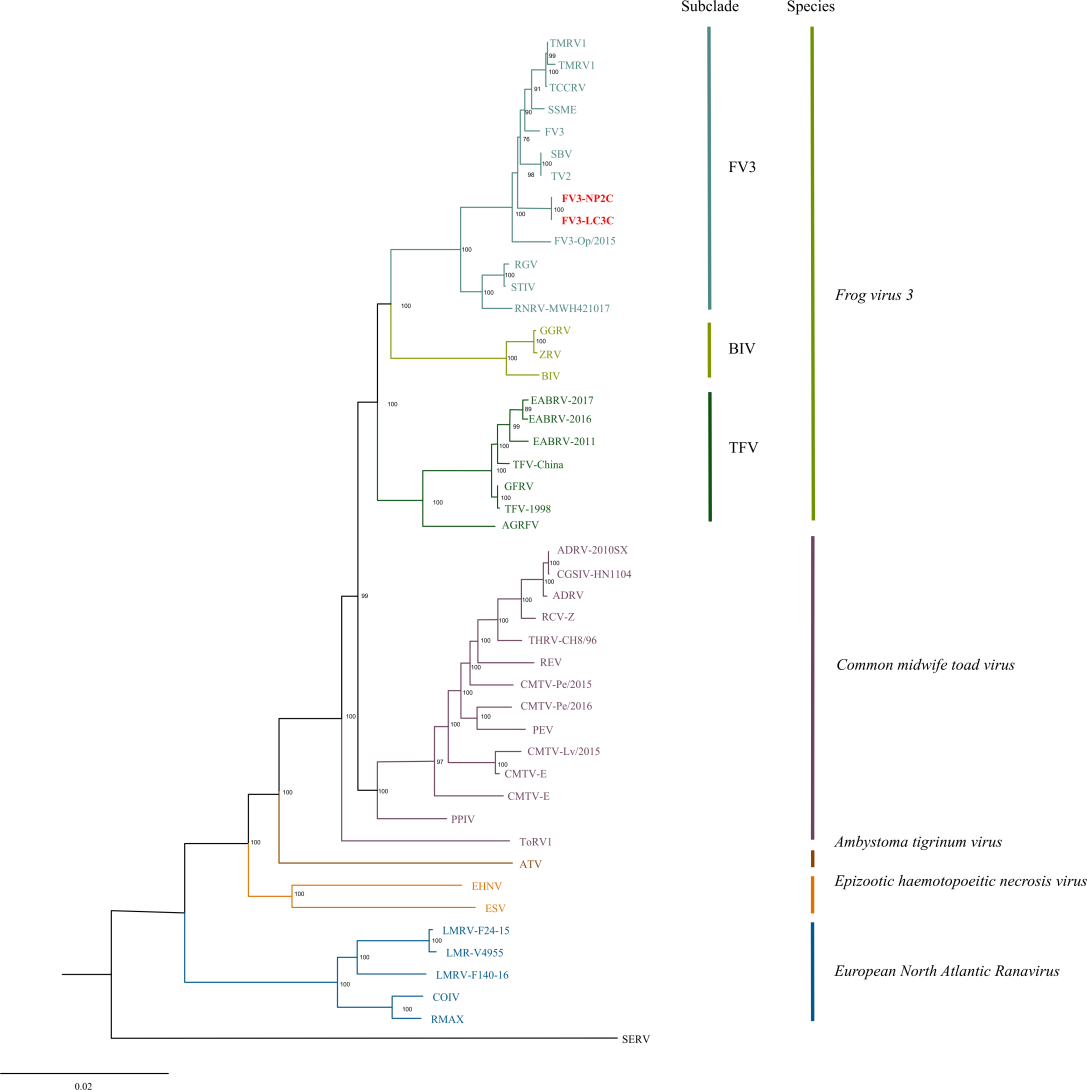
Maximum likelihood (ML) phylogram depicting the relationships of new isolates FV3-LC3C and FV3-NP2C to 44 ranaviruses, based on the concatenated genome-wide LCB alignments. New isolates are shown in red. ML analysis was performed in IQ-TREE (http://iqtree.cibiv.univie.ac.at) with default parameters and 1,000 bootstrap replicates. The bootstrap values are provided at each node. The tree was rooted with Short-finned eel ranavirus (SERV). See Table 1 for virus abbreviations.

Virus isolation was attempted by inoculating the homogenized spleen tissues, collected from NP2C and LC3C, onto EPC cells as previously described (4). After the cytopathic effects were observed in 90% of the EPC monolayer, DNA was extracted from clarified spent media using a DNeasy Blood and Tissue kit (Qiagen). DNA libraries were prepared using the tagmentation-based and PCR-based Illumina DNA Prep kit (Illumina) and custom IDT 10 bp unique dual indices. Genome sequencing was performed on an Illumina NovaSeq X Plus sequencer using 10B Reagent Kit (300 cycles) and generated 51,642,838 and 50,826,372 reads at an average read length of 133 and 135 bp for NP2C and LC3C, respectively. *De novo* assembly of the untrimmed paired-end reads in SPAdes v3.12.0 (5), using default parameters, produced contiguous consensus sequences of 106,988  bp with G + C contents of 55.1% and an average coverage of 44,482 and 45,074 reads/nucleotide for NP2C and LC3C, respectively. The full genomes of the two isolates were annotated with the Genome Annotation Transfer Utility (6), using Stickleback virus isolate 1096 (GenBank accession number MZ514903) as the reference genome. Ninety-five putative open reading frames (ORFs) were predicted for both isolates. Comparative genomic analysis revealed that these two ranaviruses were nearly identical, except for nucleotide substitutions at positions 21,127 and 22,271 in NP2C as compared to LC3C genome, resulting in non-synonymous changes in ORFs 15 (hypothetical protein) and 17 (hypothetical protein), respectively. The genome-wide locally colinear block (LCB) alignments of 44 fully sequenced ranaviruses were generated in Mauve v2.4.0 (7) using default parameters (Table 1). The LCB alignments were then concatenated in Geneious v10.2.6 (8) and used in maximum likelihood (ML) analysis in IQ-Tree v1.6.12 (9) with default parameters and 1,000 bootstrap replicates. The resulting ML tree revealed that the viruses isolated from LC3C and NP2C as strains of the species *Frog virus 3*, hereafter referred to as Frog virus 3 isolate LC3C (FV3-LC3C) and Frog virus 3 isolate NP2C (FV3-NP2C), respectively (Fig. 1).

**TABLE 1:** Virus species, isolate name, abbreviations, and GenBank accession numbers of the ranaviruses used in the phylogenetic analyses

Virus species	Isolate name (abbreviation)	GenBank accession number
*Frog virus 3*	Frog virus 3 (FV3)	AY548484
	Frog virus 3 isolate SSME (SSME)	KJ175144
	Trioceros melleri ranavirus 1 (TMRV1)	MG953519
	Trioceros melleri ranavirus 2 (TMRV2)	MG953520
	Terrapene carolina carolina ranavirus (TCCRV)	MG953518
	Frog virus 3 isolate Op/2015/Netherland (FV3-Op/2015)	MF360246
	Rana nigromaculata ranavirus (RNRV)	MG791866
	Soft-shelled turtle iridovirus (STIV)	EU627010
	Rana grylio iridovirus (RGV)	JQ654586
	Stickleback virus isolate 1096 (SBV)	MZ514903
	Tadpole virus 2 (TV2)	MZ514904
	Tiger frog virus (TFV-China)	AF389451
	Tiger frog virus isolate AV9803 (TFV-1998)	MT512504
	Tiger frog virus isolate F0207 (TFV-F0207)	MT512501
	Tiger frog virus isolate F2112 (TFV-F2112)	MT512503
	Tiger frog virus isolate D11-067 (TFV-D11-067)	MT512498
	Tiger frog virus isolate VD-16-006 (TFV-VD-16-006)	MT512499
	Tiger frog virus isolate VD-17-007 (TFV-VD-17-007)	MT512500
	Tiger frog virus isolate D03-034 (TFV-D03-034)	MT512497
	Tiger frog virus isolate D2008 (TFV-D2008)	MT512502
	Bohle iridovirus (BIV)	KX185156
	Zoo ranavirus isolate 040414 (ZRV)	MK227779
	German gecko ranavirus (GGRV)	KP266742
*Ambystoma tigrinum virus*	Ambystoma tigrinum virus (ATV)	AY150217
*Epizootic haematopoietic necrosis virus*	Epizootic haematopoietic necrosis virus (EHNV)	FJ433873
	European sheatfish virus (ESV)	JQ724856
*Common midwife toad virus*	Common midwife toad virus (CMTV-E)	JQ231222
	Common midwife toad virus (CMTV-NL)	KP056312
	Testudo hermanni ranavirus (THRV-CH8/96)	KP266741
	Tortoise ranavirus isolate 1 (ToRV1)	KP266743
	Andrias davidianus ranavirus (ADRV)	KC865735
	Andrias davidianus ranavirus (ADRV-2010SX)	KF033124
	Chinese giant salamander iridovirus (CGSIV-HN1104)	KF512820
	Common midwife toad virus (CMTV-Lv/2015)	MF004272
	Common midwife toad virus (CMTV-Pe/2015)	MF125269
	Common midwife toad virus (CMTV-Pe/2016)	MF125270
	Rana catesbeiana virus isolate RC-Z (RCV-Z)	MF187210
	Rana esculenta virus (REV)	MF538628
	Pelophylax esculentus virus (PEV)	MF538627
	Pike-perch iridovirus (PPIV)	KX574341
*European North Atlantic Ranavirus*	Lumpfish ranavirus isolate F140-16 (LMRV-F140-16)	MH665359
	Lumpfish ranavirus isolate F24-15 (LMRV-F24-15)	MH665358
	Lumpfish ranavirus isolate V4955 (LMRV-V4955)	MH665360
Unclassified	Ranavirus maximus (Rmax)	KX353311
	Cod iridovirus (CoIV)	KX574343
	Short-finned eel ranavirus (SERV)	MH665360

This study confirms that the two FV3 strains isolated from amphibian species of different orders during a natural disease outbreak are nearly identical. Understanding the role of FV3 in the decline of frogs and salamanders is a pressing conservation issue, as both species impacted here have experienced severe population declines and are listed as species of conservation concern by state or federal agencies (10, 11).

## Data Availability

The complete genome sequences of FV3-LC3C and FV3-NP2C have been deposited in GenBank under accession numbers PP179901 and PP179900, respectively. Raw sequence data for FV3-LC3C and FV3-NP2C have been deposited in the Sequence Read Archive under accession numbers SRR27645745 and SRR27645746, respectively.
